# Comprehensive Pan-Cancer Analyses of Immunogenic Cell Death as a Biomarker in Predicting Prognosis and Therapeutic Response

**DOI:** 10.3390/cancers14235952

**Published:** 2022-12-01

**Authors:** Yuan Wang, Yongbiao Huang, Mu Yang, Yulong Yu, Xinyi Chen, Li Ma, Lingyan Xiao, Chaofan Liu, Bo Liu, Xianglin Yuan

**Affiliations:** Department of Oncology, Tongji Hospital, Tongji Medical College, Huazhong University of Science and Technology, Wuhan 430030, China

**Keywords:** immunogenic cell death, pan-cancer, single-cell, tumor microenvironment, immunotherapy, IGF2BP3

## Abstract

**Simple Summary:**

Immunogenic cell death (ICD) is an important mechanism underlying anti-cancer therapy response by activating the immune system. However, the landscape and predictive value of ICD among cancers remain to be elucidated. In this study, we carried out a comprehensive analysis integrating genomic, proteomic and epigenetics data across 33 cancer types, 31 normal tissue types and 1406 cancer cell lines. We found that the expression level of ICD-related genes was regulated by methylation and transcriptional regulators. We figured out the most valuable ICD-related markers in predicting prognosis in cancer patients and developed a model for practical use. We also defined the ICD score and found that it was a reliable marker in predicting survival, chemotherapy and immunotherapy across cancer patients. Further exploration using single-cell RNA-seq data indicated that T cell might remodel the tumor microenvironment by turning a “cold” tumor into a “hot” one in an ICD-dependent manner. Moreover, we also discovered several ICD-related therapeutic targets including IGF2BP3 which might benefit cancer patients who could hardly respond to immunotherapy.

**Abstract:**

Immunogenic cell death (ICD), a form of regulated cell death, is related to anticancer therapy. Due to the absence of widely accepted markers, characterizing ICD-related phenotypes across cancer types remained unexplored. Here, we defined the ICD score to delineate the ICD landscape across 33 cancerous types and 31 normal tissue types based on transcriptomic, proteomic and epigenetics data from multiple databases. We found that ICD score showed cancer type-specific association with genomic and immune features. Importantly, the ICD score had the potential to predict therapy response and patient prognosis in multiple cancer types. We also developed an ICD-related prognostic model by machine learning and cox regression analysis. Single-cell level analysis revealed intra-tumor ICD state heterogeneity and communication between ICD-based clusters of T cells and other immune cells in the tumor microenvironment in colon cancer. For the first time, we identified IGF2BP3 as a potential ICD regulator in colon cancer. In conclusion, our study provides a comprehensive framework for evaluating the relation between ICD and clinical relevance, gaining insights into identification of ICD as a potential cancer-related biomarker and therapeutic target.

## 1. Introduction

Immunogenic cell death (ICD), first proposed to evaluate the efficacy of cytotoxic anticancer drugs [1,2], is one form of regulated cell death upon oxidative-endoplasmic reticulum stress. In the process of ICD, dead cells release damage-associated molecular patterns (DAMPs) to elicit immune responses [3,4]. Widely accepted DAMPs include annexin A1 (ANXA1), high-mobility group box 1 (HMGB1), heat-shock proteins, calreticulin (CALR), type I interferons (IFNs) and ATP [3,5]. Release of DAMPs results in the stimulation of pattern recognition receptors (PRRs) on dendritic cells (DCs), macrophages and natural killer cells (NK cells), which subsequently stimulates and facilitates the priming of tumor-specific CD8+ T cells [6,7,8].

ICD is important in anti-infectious and anticancer immunity [9]. Accumulating evidence revealed that ICD could be induced by anticancer treatments including chemotherapy [10], photodynamic therapy [11,12,13,14], photothermal therapy [15], extracorporeal photochemotherapy [16], radiotherapy [17,18], oncolytic virotherapy [19,20] and targeted agents [21,22,23,24,25]. Combination use of chemotherapy, photodynamic therapy and immunotherapy could orchestrate to initiate powerful antitumor immune response in an ICD-dependent manner [14].

However, the role of ICD in the tumor microenvironment (TME) seems controversial in different tumor types. ICD of malignant cells could alter the TME by generating potential neoantigens, playing either an immunostimulatory or immunosuppressive role [26,27]. A previous large-scale meta-analysis among non-small cell lung cancer (NSCLC), breast cancer (BRCA) and ovarian cancer (OV) patients confirmed the ICD signature was a reliable prognosis predictor [28]. The ICD-related genes they identified encoded molecules which acted as ICD-associated danger signals or danger signal-degraders, participated in ICD execution as danger signaling components, functioned as innate immune effectors or T cell effectors associated with ICD and participated in the purinergic receptor-inflammasome-interleukin1β axis, toll-like receptor signaling, or T cell infiltration pattern associated with ICD. However, knowledge about the predictive power of ICD at a pan-cancer level seldom exists. A comprehensive understanding of the expression and genetic alteration landscape of ICD biomarkers is necessary to elucidate its role in modeling TME and determining pharmacogenomic characteristics.

**Figure 1:**
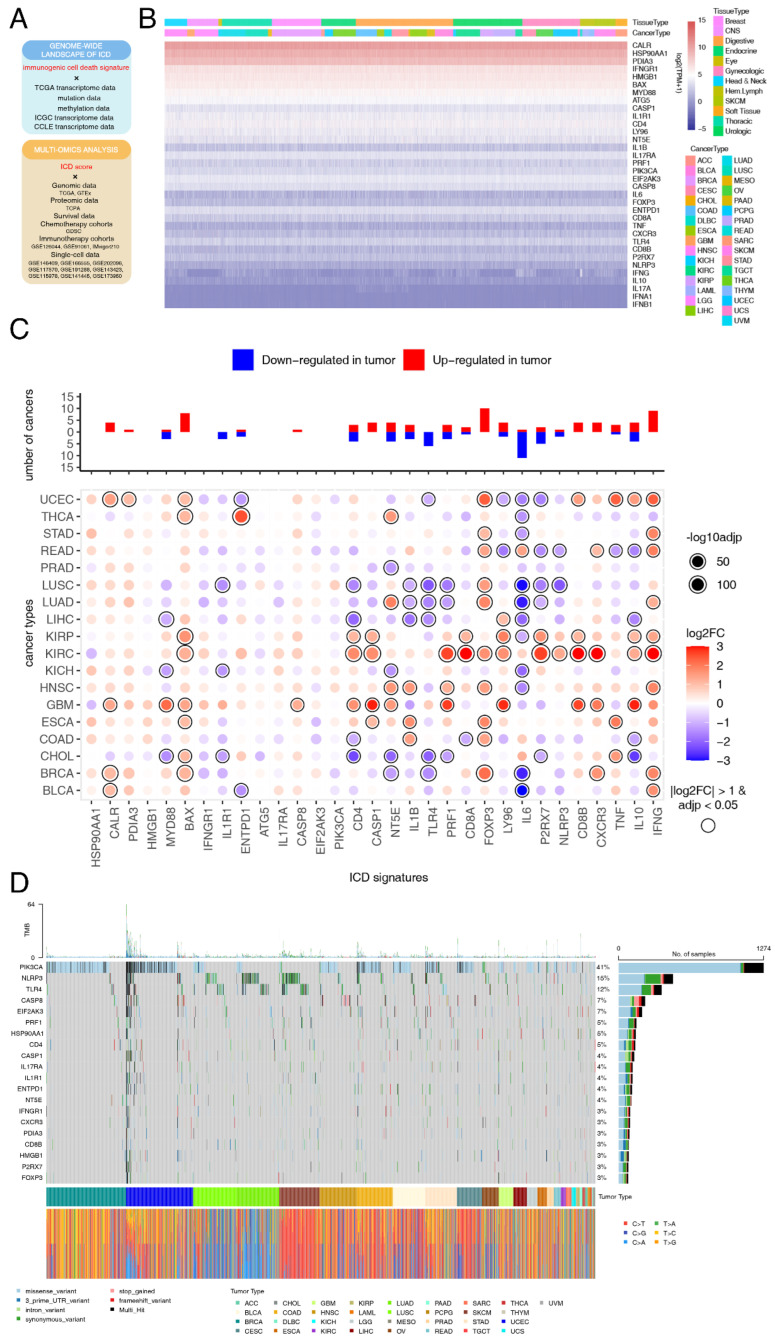
Landscape of expression and mutagenesis of immunogenic cell death (ICD) signatures in pan-cancers. (**A**) An overview of the data used in the study. Multi-omics data was used to depict the ICD landscape across cancers and normal tissues. Bulk and single-cell RNA-sequencing data were used to figure out the role of ICD in overall survival (OS), anti-cancer therapy response and tumor microenvironment (TME) remodeling. (**B**) A comprehensive heatmap illustrating the expression pattern of ICD-related genes across 33 solid cancer types from The Cancer Genome Atlas (TCGA). (**C**) Bar plot at the top showing the total number of cancer types in which each of the ICD-related genes was upregulated or downregulated. The heatmap at the bottom shows the fold-change of ICD-related genes expression level in cancerous tissues compared with normal tissues. (**D**) Onco-plot showing the distribution of the 20 most frequently mutated ICD-related genes in different cancer types.

Based on these factors, the current study was designed to explore the relation between ICD and cancer (Figure 1A). Briefly speaking, we first analyzed the expression and genomic mutagenesis profiles of ICD-related genes at a pan-cancer level. Then, we defined a computational metric to calculate ICD scores. Comprehensive analysis integrating multi-omics data revealed that the ICD score was a good predictor of tumor proliferation, metastasis and microenvironment. In the context of clinical relevance, ICD scores were powerful in predicting chemotherapy and immunotherapy responses and patient survival at both bulk and single-cell RNA-sequencing (RNA-seq) level. In summary, our integrated analyses provided a insight into the role of ICD in different hallmarks of cancer, shedding light on the development of the ICD-related prognostic biomarker and therapeutic target.

## 2. Materials and Methods

### 2.1. Data and Resources

Transcriptome profiling data, somatic mutation data, DNA methylation data and relevant clinical information of 33 tumor types were downloaded from The Cancer Genome Atlas (TCGA) database using the R package “TCGAbiolinks”. Transcriptome and clinical data of 9920 patients in the International Central Gospel Church (ICGC) dataset was downloaded from the ICGC Data Portal (https://dcc.icgc.org/, accessed on 5 July 2022). Transcriptome profiling data of 31 normal tissue types and related information in the Genotype-Tissue Expression (GTEx) database were obtained from UCSC Xena (http://xena.ucsc.edu/public/, accessed on 7 July 2022). Gene expression profiles and relevant information of 1406 cancer cell lines (CCLs) from 29 tissue types were downloaded from the Cancer Cell Line Encyclopedia (CCLE) database (https://sites.broadinstitute.org/ccle/, accessed on 11 July 2022). Protein expression data of 33 tumor types were downloaded from The Cancer Proteome Atlas (TCPA) database (https://www.tcpaportal.org/tcpa, accessed on 14 July 2022). Gene expression data of different TCGA cohorts were merged and then batch effects were removed via Combat method in the R package “sva”. Expression profiles and corresponding clinical data of immunotherapy cohorts were retrieved from the Gene Expression Omnibus (GEO) database (https://www.ncbi.nlm.nih.gov/geo/, accessed on 23 July 2022) and the R package “IMvigor210CoreBiologies” [29]. Single cell-RNA (sc-RNA) sequencing datasets were downloaded from the GEO database. Fragments Per Kilobase of exon model per Million mapped fragments (FPKM) and raw counts data were transformed to transcripts per million (TPM) followed by log-transformation. Detail information of the clinical cohorts used in this study is listed in Table S1.

### 2.2. ICD Score Calculation and ICD Score Based Clustering

ICD-related genes were collected from published literature (Table S2) [28]. The “GSVA” and “GSEABase” R packages were utilized to calculate the ICD scores based on sc- and bulk RNA-seq data. For sc-RNA sequencing datasets, the ICD score of each cell was calculated using the function enrichIt in the R package “escape”.

Unsupervised consensus clustering was carried out using the function ConsensusClusterPlus in the R package “ConsensusClusterPlus”. Data was normalized with the z-score method before the clustering process. Parameters were set as follows: maxK = 6, reps = 50, pItem = 0.8, pFeature = 0.8, clusterAlg = “hc” and distance = “pearson”. Number of clusters selected was based on both clustering results and clinical significance.

### 2.3. Differential Expression Analysis

Gene differential expression analysis based on the counts matrix was performed in 18 cancer types with number of normal samples ≥ 5 using the R package “DESeq2” [30]. Wilcoxon’s rank-sum test was used to evaluate the difference in transcriptional expression between normal tissues and cancerous. Genes with |log2(fold-change)| > 1 and *p*-adjusted < 0.05 were identified as differentially expressed genes (DEGs). For differential protein expression analysis between the ICD-CB and ICD-CC subtype across 33 cancer types, we followed a similar pipeline with the R package “limma” [31].

### 2.4. Mutation and Methylation Analysis

Mutation annotation format (MAF) files identified by MuTect2 were visualized in an onco-plot using the R package “maftools”. For DNA damage repair (DDR) response mutation analysis, a specific pathway would be considered mutated if any gene involved was non-silently mutated. Spearman correlations were calculated between gene expression level and corresponding methylation level. 

### 2.5. Transcriptome Analysis

To identify main regulators of ICD-related genes, the iRegulon plugin in the Cytoscape software was applied [32]. Following parameters were used for analysis: 0.05 for minimum identity between orthologous genes, enrichment score ≥ 4.0 and 0.001 for maximum false discovery rate on motif similarity.

### 2.6. Gene Set Variation Analysis (GSVA) and Pathway Analysis

GSVA [33] was utilized to find out signaling pathways which the DEGs involved across TCGA cancer types based on the gene sets and transcriptome data retrieved from Molecular Signatures Database (MSigDB) [34]. Epithelial-mesenchymal transition (EMT) score was calculated based on a previously published literature [35]. EMT score was established with the GSVA algorithm by separately inferring the activities of up-regulated and down-regulated EMT signature genes in individual samples and then calculating the differences between these two activities. DEGs between the high ICD score and low ICD score group in the TCGA-COAD cohort were further analyzed with the online tool Oncobox pathway databank (OncoboxPD) (https://open.oncobox.com/, accessed on 10 November 2022) [36], a collection of seven databases measuring the pathway activation levels (PALs).

### 2.7. Survival Analysis

To identify the relationship between ICD and survival at a pan-cancer level, the survfit function in the R package “survival” was used to calculate the hazard ratio (HR) of ICD score. ICD score was considered as a risky factor if HR > 1 and *p* < 0.05 and as a protective one if HR < 1 and *p* < 0.05. The survival differences between the high-ICD score and low-ICD score group were further visualized by Kaplan-Meier survival curves with the R packages “survival” and “survminer”.

### 2.8. Development of Prognostic Model Based on ICD-Related Genes

Four machine learning algorithms, Boruta [37], eXtreme Gradient Boosting (XGBoost) [38], support vector machine (SVM) [39] and Random Forest [40], were used to screen out the most valuable ICD-related genes for predicting prognosis at a pan-cancer level. Next, univariate Cox regression analysis were used to screen out the prognostic ICD-related genes considering important clinical characteristics including age, gender, T stage, N stage and M stage. A multivariate Cox regression model was further constructed with estimated regression coefficients. The R packages “timeROC” and “survivalROC” were utilized to estimate the area under curve (AUC) in different cohorts.

### 2.9. Immune Landscape Analysis

Estimation of STromal and Immune cells in MAlignant Tumor tissues using Expression data (ESTIMATE) algorithm was used to evaluate stromal score, immune score, ESTIMATE score and tumor purity [41]. Absolute abundances of 22 immune cell types across TCGA cohorts were inferred by CIBERSORT (https://cibersort.stanford.edu/, accessed on 26 July 2022) [42], with LM22 as the standard annotation file. Spearman correlation between the ICD score and immune cell abundance of each cancer type was calculated.

### 2.10. Immunotherapy Response Analysis

Gene expression data and relevant clinical information of 376 patients receiving immunotherapy were integrated from 3 cohorts. The R package “pROC” was used to estimate the AUC of each cohort. Non-synonymous somatic mutations were used to calculate tumor mutation burden (TMB). Cytolytic activity (CYT) score was defined as the geometric mean of the transcription level of perforin 1 (PRF1) and granzyme A (GZMA) [43]. Spearman correlations between the ICD score and the transcription level of PDL1 and TCLA4 in each cancer type were calculated respectively.

### 2.11. Drug Sensitivity Estimation

Drug sensitivity data of CCLs were downloaded from the Genomics of Drug Sensitivity in Cancer (GDSC) database [44]. The IC50 value for each compound was estimated using the R package “oncoPredict”. The Spearman correlation coefficient (SCC) between ICD score and IC50 for each cancer type-drug pair was calculated. Drugs with |SCC| > 0.15 and *p* < 0.05 were identified.

### 2.12. Single-Cell Data Analysis

For single-cell data analysis, raw count matrix was analyzed with the R package “Seurat”. A total of 50,715 cells were analyzed and low-quality cells (UMI count > 1000, number of genes detected per UMI between 200 and 6000, mitochondrial percent < 10%) were removed. Doublets were predicted with the R package “DoubletFinder” [45] and were excluded in subsequent analysis. Batch effects across different samples were eliminated with the Harmony method [46]. The FindNeighbors and FindClusters function were used to generate clustering results at different resolutions ranging from 0.2 to 1.2. The optimum resolution was chosen based on clustering stability with the R package “clustree” [47]. Cell clusters were manually annotated based on markers retrieved from previously published literature and clusters which did not express known markers were excluded in the subsequent analysis. After following the standard pipeline, 24 cell clusters were identified and mapped into 4 main cell types based on canonical markers (Table S3).

For the analysis of the dataset GSE166555 containing 30,642 single cells, clustering procedures for immune cells, epithelial cells and T cells were the same as above. Briefly, we obtained 24 clusters (resolution = 1.2) after quality-control filtering (Figure S1A) and roughly classified them into 3 main cell types using canonical markers (Table S3). For epithelial cells, we identified 11 clusters (resolution = 0.4) (Figure S1B) and classified them into iCMS2 and iCMS3 subtypes based on 715 iCMS marker genes (Table S3). As for immune cells, we identified 18 clusters (resolution = 0.4) (Figure S1C) and classified these into ten subtypes based on cell marker genes from a previous study (Table S3) [48]. For T cells, four cell types were identified from seven clusters (Figure S1D). DEGs among different clusters of epithelial cells were identified using the function FindMarkers. Significant DEGs with *p*-adjusted < 0.05 and |log2(fold-change)| > 1 were further enriched in the Reactome database using the R package “ReactomePA”. CellPhoneDB was used for cell-cell communication analysis [49]. Significant interaction pairs were chosen with *p* < 0.05.

### 2.13. Cell Culture and Transfection

The normal human colon cell line NCM460 and human colon CCLs HCT116, HT29, LoVo and SW480 were obtained from the oncology laboratory of Tongji Hospital, Wuhan, China. HCT116 cells were cultured in McCoy’s 5A medium (Procell, Wuhan, China) containing 10% fetal bovine serum (FBS, Gibco, New York, NY, USA) while the other colon cells were cultured in DMEM medium (HyClone, Logan, UT, USA) containing 10% FBS. Cells were maintained at 37 °C supplemented with 5% CO2 in the incubator. IGF2BP3 siRNA and negative control (NC) were synthesized by General Biol (Anhui, China) and transfected into cells using LipoFectMax (ABP Biosciences, Rockville, WA, USA) according to the manufacturer’s instructions. The sequences of IGF2BP3-siRNAs and NC were listed in Table S4.

### 2.14. Western Blot Analysis

Total cellular protein was extracted with RIPA lysis buffer (Servicebio, Wuhan, China) containing phosphorylase and protease inhibitors (Servicebio, Wuhan, China) and then denatured by mixing 5× loading buffer (Servicebio, Wuhan, China) and boiling for 10 min. Then the denatured protein was separated by electrophoresis in 10% SDS-PAGE gels and transferred to PVDF membranes (Sigma, Darmstadt, Germany). The membranes were blocked in 5% BSA (Servicebio, Wuhan, China) for 1 h at room temperature and then incubated with the following primary antibodies: IGF2BP3 (Abclonal, Wuhan, China, CAT# A6099, 1:1000) and GAPDH (Huabio, CAT# ET1601-4, 1:5000) at 4 °C overnight. Then the membranes were washed with TBST 10 min for three times and incubated with the secondary antibody (Promotor, Wuhan, China, CAT# HA1005, 1:5000) at room temperature for 1 h. Finally, the proteins were visualized with West Pico plus Chemiluminescent Substrate (Thermo Fisher Scientific, Carlsbad, CA, USA).

### 2.15. Quantitative Real-Time Polymerase Chain Reaction (qRT-PCR)

Total RNA from cells was extracted using RNA isolator Total RNA Extraction Reagent (Vazyme, Nanjing, China) and reverse transcribed with HiScript II Q RT SuperMix (Vazyme, Nanjing, China). Then qRT-PCR was carried out in Real-Time PCR System (7900HT, Applied Biosystems, Carlsbad, CA, USA) with ChamQ Universal SYBR qPCR Master Mix (Vazyme, Nanjing, China). The mRNA level of interested genes was normalized to that of the reference gene GAPDH. Primer sequences were listed in Table S4.

### 2.16. Statistical Analysis

Student’s *t*-test was performed for normally distributed data and one-way analysis of variance (ANOVA) was used for comparisons between two groups with non-normally distributed data. Two-sided Kruskal-Wallis test was used for comparisons among more than two groups with non-normally distributed data. The log-rank test was used for survival analysis. *p* value was adjusted with the false discovery rate (FDR) for multiple testing correction in multiple comparisons. All statistical analyses were performed in the R software (Vienna, Austria, version 4.2.0) and the GraphPad Prism software (San Diego, CA, USA, version 8.0.1) and *p*-value < 0.05 was considered as statistically significant.

## 3. Results

### 3.1. Landscape of ICD-Related Genes Expression and Mutagenesis in Pan-Cancers

To depict the pan-cancer genomic landscape of the 34 ICD-related genes, a comprehensive heatmap was generated to display the expression profile in a total of 10,327 TCGA samples across 33 solid cancer types (Figure 1B). We observed that the expression pattern of ICD-related genes was consistent among different cancer types. Among all of the ICD-related genes, CALR showed the highest expression in pan-cancers, while IFNB1 showed the lowest expression. These findings were verified across 1076 CCLs originated from 26 different tissue types (Figure S2A). Spearman correlation analysis was performed among all the ICD-related genes. We found that most pairs exhibited significantly positive correlations, especially CD4 with the other ICD-related genes (Figure S2B). We then compared the expression of ICD-related genes between primary tumors and adjacent normal tissues (ANTs) across 18 cancers. Overall, 24 (70.6%) of the ICD-related genes were differentially expressed in at least one cancer type (Figure 1C). Several ICD-related genes were consistently regulated in multiple cancer types. We discovered that FOXP3 had relatively higher expression level in cancerous tissues than ANTs, while IL6 had relatively lower expression. However, the role of cancer cell FOXP3 in tumorigenesis is conflicting [50,51,52,53,54,55,56,57,58] and still warrants further investigation.

To explore the mutation landscape of ICD-related genes at a pan-cancer level, an onco-plot was built to display the 20 most frequently mutated ICD-related genes among different cancer types (Figure 1D). In summary, ICD-related genes were altered in 2666 (85.48%) of 3119 samples. The most frequently mutated ICD-related genes at the pan-cancer level was PIK3CA (41%) with missense variation. PIK3CA mutation is likely to function as an oncogene in human cancers [59]; however, its role in ICD had not been systematically investigated.

To figure out mechanisms contributing to the expression profile of ICD-related genes across cancers, methylation and transcriptional regulators were further analyzed. DNA methylation could prevent gene activation in tumorigenesis [60]. Analysis between the transcription level and methylation level of ICD-related genes revealed that methylation played a complex role in the expression of ICD-related genes in pan-cancers (Figure S2C). Methylation was significantly negatively correlated with the transcription level of CD4 and LY96 in almost all the cancer types, contributing partially to the low expression of these genes. Transcriptional regulation is an essential component of tumor progression and metastasis [61]. Master transcriptional regulators of the ICD-related genes predicted by iRegulon were Ets1, YY2 and JDP2 (Figure S2D, Table S5). Ets1 [62], YY2 [63] and JDP2 [64] all contribute to cancer cell proliferation, invasiveness, EMT, drug resistance and neo-angiogenesis by regulating gene transcription. Targeting these transcriptional regulators might be potential anti-tumor therapies through an ICD-dependent manner.

### 3.2. Molecular and Clinical Characteristics of Clusters Based on ICD Score

**Figure 2:**
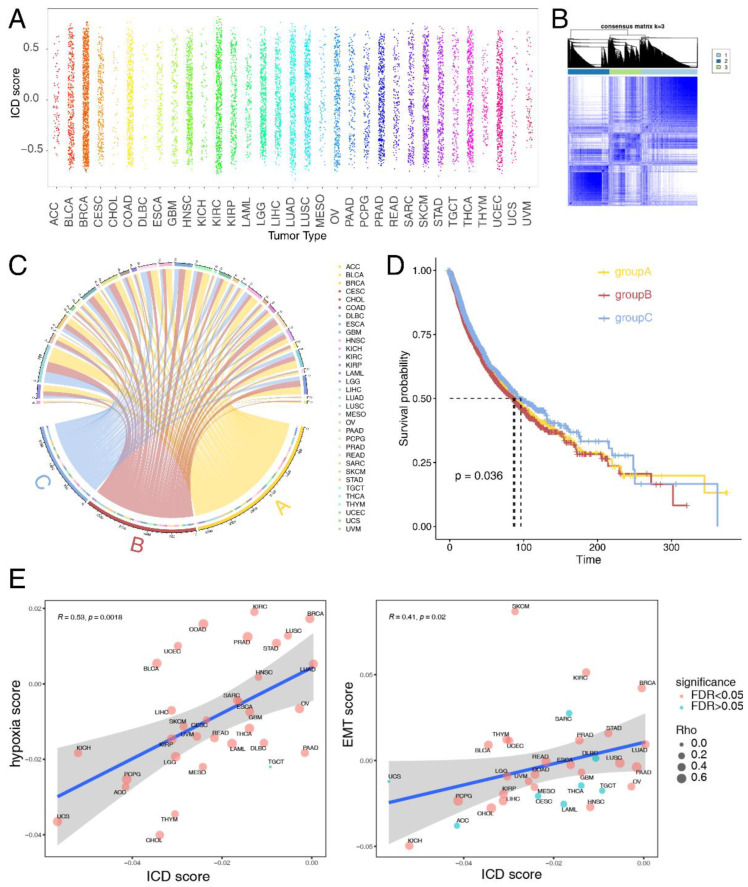
Clinical and functional characteristics in different ICD-subtypes. (**A**) Average ICD scores in different cancer types. (**B**) The consensus matrix showing the unsupervised clustering result of patients in the TCGA cohort based on ICD score. (**C**) The chord diagram showing allocation of TCGA patients in cluster A (ICD-CA), cluster B (ICD-CB) and cluster C (ICD-CC). (**D**) The Kaplan-Meier curve of OS among the ICD-CA, ICD-CB and ICD-CC subtypes at a pan-cancer level. (**E**) The Spearman correlation between ICD score and hypoxia score (upper panel) and EMT score (lower panel). Size of the point is proportional to the Spearman correlation coefficient and color of the point represents the significance of the Spearman correlation test. Time is measured in days.

ICD scores were calculated as previously mentioned to systematically depict ICD levels across 33 cancer types in the TCGA database (Figure 2A). We observed no significant difference in the distribution of ICD scores across distinct cancer types, which was consistent with the expression landscape of ICD signatures we found previously. This indicated that tissue type might not be an essential determinant contributing to ICD state of cancer patients; instead, a few high-expressed genes including CALR, HSP90AA1 and PDIA3 could regulate the process of ICD at a pan-cancer level. Consistent ICD score distribution profile was also observed across 31 normal tissue types in the GTEx database (Figure S3A). Based on unsupervised clustering in all cancer patients, we identified three distinct clusters, namely cluster A (ICD-CA), cluster B (ICD-CB) and cluster C (ICD-CC) (Figure 2B). An overview of the distribution of all the cancer patients is shown in Figure 2C. We then compared patient overall survival (OS) among the three clusters to evaluate whether ICD score could predict prognosis. As shown in Figure 2D, a significant OS difference existed among these three clusters (*p* = 0.036). Patients in the ICD-CB had the shortest OS, while patients in the ICD-CC subtype had the longest. Next, we applied differentially expressed proteins analysis between the ICD-CB and the ICD-CC subtype across cancers (Figure S3B). We found that IGFBP2 was significantly upregulated in the ICD-CB subtype compared with the ICD-CC subtype in pheochromocytoma and paraganglioma (PCPG).

Furthermore, we found that ICD affected numerous cancer-related pathways. Tumor hypoxia [65,66] and EMT [67] are major hallmarks of tumors. Previous study found that tumor hypoxia might drive aggressive molecular features across cancers [65]. Thus, we calculated Spearman correlations between ICD score and these indicators. We observed a robustly positive correlation between ICD score and tumor hypoxia score in nearly all of the cancer types except testicular cancer (TGCT) (R=0.53, *p* = 0.0018) (Figure 2E). Moreover, a significant correlation was also observed between ICD score and EMT score in most cancer types (R = 0.41, *p* = 0.02) (Figure 2E). 

### 3.3. Construction and Validation of Prognostic Model Based on ICD-Related Genes

To construct a prognostic model based on ICD-related genes, cancer patients were randomly allocated into the training set and the test set at a ratio of 7:3 and four machine learning algorithms, Boruta (Figure S4A), XGBoost (Figure S4B), SVM and Random Forest (Figure S4C), were applied to identify the two most valuable ICD-related genes from 34 ICD-related genes (Table S2). Then univariate Cox proportional hazards regression analysis was performed to identify the most reliable predictors from the two ICD-related genes and other clinical characteristics including age, sex and tumor stage. We found that IL6 (*p* = 0.03), age (*p* < 0.001), gender (*p* < 0.001), T stage (*p* < 0.001), N stage (*p* < 0.001) and M stage (*p* < 0.001) were significantly related with OS (Figure S4D). A prognostic model based on these predictors was finally constructed using the multivariate Cox regression analysis (Figure S4E). Patients with high ICD-related prognostic signature scores had significantly decreased OS in the TCGA cohort (Figure S4F). The time-dependent ROC curve showed that the AUC values reached 0.708, 0.725 and 0.862 at 5, 10 and 20 years, respectively (Figure S4G). The prognostic model was further validated in the ICGC cohort to evaluate its robustness. Consistent with the results in the TCGA cohort, patients with high scores were characterized by bad OS in the ICGC cohort (Figure S4H). Besides, the AUC values of the time-dependent ROC curves at 5, 10 and 20 years reached 0.642, 0.8 and 0.852, respectively (Figure S4I). Altogether, these results confirmed the predictive value of the ICD-related prognostic model we constructed.

### 3.4. Immune Landscape Associated with ICD Score

**Figure 3:**
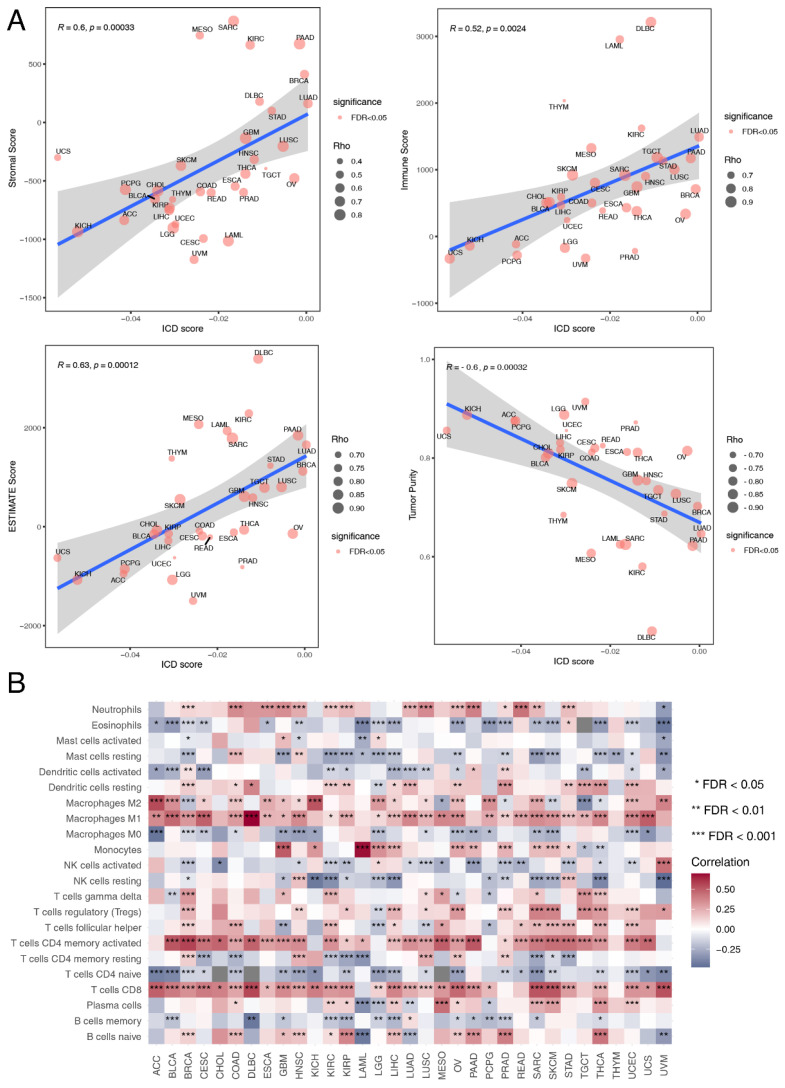
Relationship between ICD score and immune landscape at the pan-cancer level. (**A**) The Spearman correlation between ICD score and stromal score, immune score, ESTIMATE score and tumor purity respectively. Size of the point is proportional to the absolute value of Spearman correlation coefficient and color of the point represents the significance of the Spearman correlation test. (**B**) Heatmap showing Spearman correlations between ICD score and the absolute abundance of 22 immune cell types calculated by the CIBERSORT algorithm for each TCGA cancer type. * represents false discovery rate (FDR) < 0.05, ** represents FDR < 0.01, *** represents FDR < 0.001.

Tumor immunity plays a critical role in tumor growth and cancer patient prognosis [68], so we also investigated the influence of ICD on tumor immunity. Immune cell infiltration estimation with the ESTIMATE algorithm showed that tumor purity was negatively correlated with ICD score in all of the 33 cancer types (R = −0.6, *p* = 0.00032) (Figure 3A). Besides, ICD score was positively correlated with stromal score (R = 0.6, *p* = 0.00033) (Figure 3A), immune score (R = 0.52, *p* = 0.0024) (Figure 3A) and ESTIMATE score (R = 0.63, *p* = 0.00012) (Figure 3A). This indicated that ICD score could alter the proportion of stromal cells and immune cells in the TME. We further analyzed the influence of ICD on immune cell components of the TME. The correlation heatmap illustrated the relationships among ICD score and different immune cell infiltration across cancers (Figure 3B). Most immune cell abundance was significantly correlated with ICD scores in different cancer types. Macrophages M1 infiltration was strongly positively correlated with ICD scores, especially in large B-cell lymphoma (DLBC). As macrophages M1 is an inflammatory subtype [69], this indicated that ICD had the potential to turn a “cold” tumor into a “hot” one. Besides, monocyte abundance was strongly positively correlated with ICD scores in acute myeloid leukemia (LAML). These results revealed the potential role of ICD in modeling the TME.

### 3.5. Therapeutic Effect Prediction Based on ICD-Related Genes at a Pan-Cancer Level

To further investigate clinical implications of the ICD score, IC50 values of 198 compounds from GDSC were estimated for each CCL. We found that cancer patients with different ICD scores would probably benefit from a variety of chemotherapies depending on tumor types (Figure S5A).

**Figure 4:**
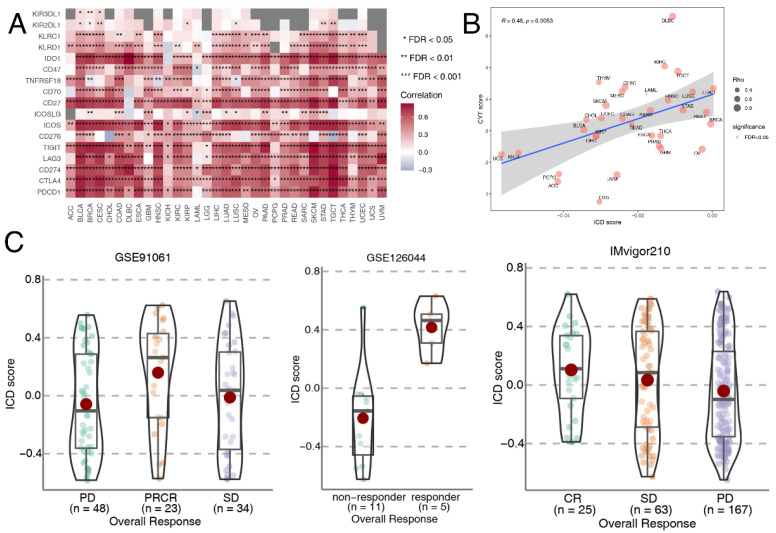
Associations of ICD scores with immunotherapy response. (**A**) Heatmap showing Spearman correlations between ICD score and canonical immune checkpoint blockade (ICB) markers in each TCGA cancer type. (**B**) The Spearman correlation between ICD score and cytolytic activity (CYT) score. Size of the point is proportional to the absolute value of Spearman correlation coefficient and color of the point represents the significance of the Spearman correlation test. (**C**) Violin plot showing distribution of ICD scores in responders and non-responders of immunotherapy. The pairwise Games-Howell test was used for *p*-value calculation. CR, complete response. PR, partial response. SD, stable disease. PD, progressive disease. * represents FDR < 0.05, ** represents FDR < 0.01, *** represents FDR < 0.001.

Next, we identified immune molecular characterizations contributing to immunotherapy response in ICD subgroups. Increased genome instability is a hallmark of cancer and the relationship between ICD and genomic variation at the pan-cancer level remains to be illustrated. TMB could predict survival after immunotherapy across multiple cancer types [70,71,72]. Thus, TMB was calculated for each tumor sample across different cancer types. We found that TMB was the highest in the ICD-CB subtype, while TMB was the lowest in the ICD-CC subtype (*p* = 0.027) (Figure S5B). The relation between the ICD score and the expression level of canonical immune checkpoint blockade (ICB) markers was also analyzed. We found that the expression level of most ICB markers positively correlated with the ICD score in almost all of the cancer types (Figure 4A). CYT score is a valuable index for assessing T-cell cytotoxicity and is a biomarker for predicting immune response [43,73]. We observed that the CYT score was positively correlated with ICD score across all of the TCGA cancer types (R = 0.48, *p* = 0.0053) (Figure 4B). Alteration of DDR genes was associated with favorable ICB outcomes in cancer patients [74]. Total mutation rates of seven DDR pathways [(base excision repair (BER), Fanconi anemia (FA), mismatch repair (MMR), homologous recombination repair (HRR), non-homologous DNA end joining (NHEJ), nucleotide excision repair (NER), trans-lesion synthesis (TLS)] were summarized in the high-ICD score and low-ICD score group across different cancer types. No significant difference was observed between the two groups (Figure S5C).

We further compared ICD scores among NSCLC, SKCM and BLCA patients with different immunotherapy outcomes. The results were consistent in the three datasets. ICD scores of responders were significantly higher than non-responders (Figure 4C). ICD scores also exhibited sound predictive power for ICB response outcomes in the cohorts GSE91061 (AUC: 0.6537, 95% CI: 0.5188-0.771) and IMvigor210 (AUC: 0.603, 95% CI: 0.5051-0.6971) (Figure S5D). These results verified ICD as a valuable biomarker in predicting immunotherapy outcomes in multiple cancer types and patients with high ICD scores might benefit more from immunotherapy.

### 3.6. Single-Cell Analyses Reveal ICD Heterogeneity and Associated Immune Signaling

**Figure 5:**
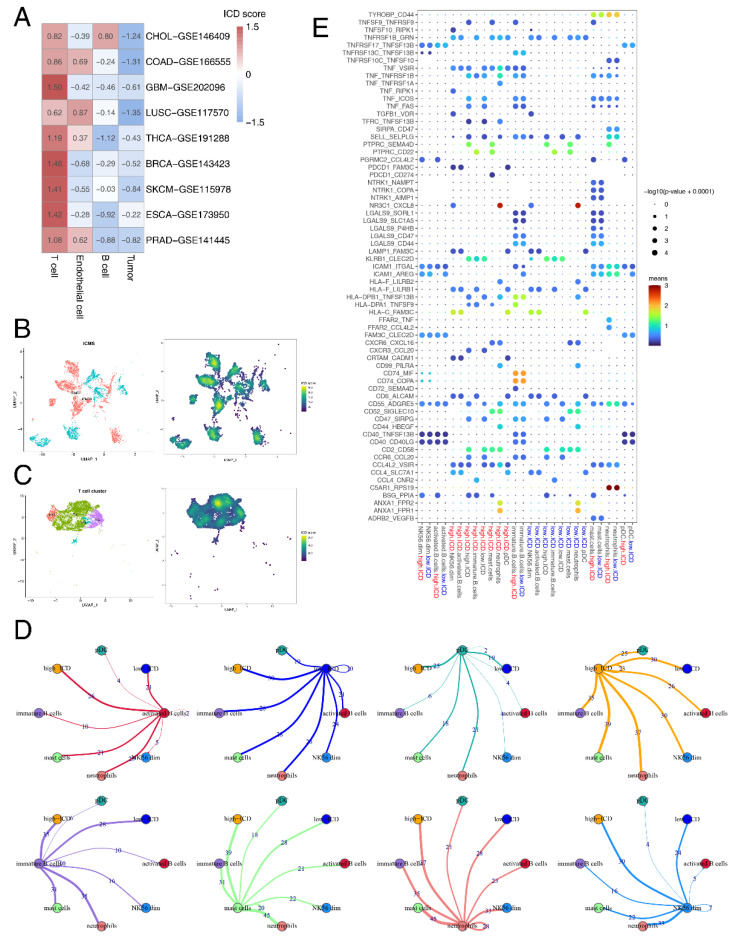
ICD heterogeneity of different cell clusters and remodeling of the TME. (**A**) Heatmap showing the average ICD score of each cell type (T cell, endothelial cell, B cell and tumor cell) in 9 single-cell datasets. (**B**) Uniform manifold approximation and projection (UMAP) plot showing 11 subtypes of epithelial cells, colored by iCMS types and ICD scores. (**C**) UMAP plot showing 7 subtypes of T cells, colored by cell types and ICD scores. (**D**) A detailed view of the ligand-receptor interaction of each cell type. Numbers indicated number of ligand-receptor pairs for each intercellular communication. (**E**) Bubble chart showing the ligand-receptor interaction landscape between ICD score-high/ICD score-low T cells (high.ICD/low.ICD) and the other cells. Ligand-expressing cells and receptor-expressing cells were shown on the x-axis; ligands and receptors were shown on the y-axis. Color denotes the average expression level of ligands and receptors in interacting cells and the bubble size represents the significance of the interaction.

To examine whether ICD scores are heterogeneous in the TME, we used nine single-cell datasets from 80 patients with different cancers. Interestingly, we observed a consistent distribution of ICD scores across cell types in different datasets (Figure 5A). The highest ICD scores were discovered in T cells, while tumor cells showed the lowest ICD scores among all the cell types. This could be attributed to the fact that the ICD-related genes mainly consisted of biomarkers related to T cell activity [28].

To explore the impact of intratumor ICD heterogeneity on the TME, we further analyzed single-cell data from 12 COAD patients. 

As previous results showed that T cells and tumor cells had significant correlation with ICD in the TME, we extracted immune cells and epithelial cells and further divided them into minor cell subpopulations. For epithelial cells, two intrinsic subtypes, iCMS2 and iCMS3, were identified based on distinct gene expression, DNA copy number and gene regulatory network in a previous colorectal cancer (CRC) single-cell study [75]. We observed that ICD scores of the iCMS2 subtype were higher than that of iCMS3 (Figure 5B). Pathway enrichment of DEGs in epithelial cells between the high-ICD score and low-ICD score group revealed the significant enrichment of immune-related pathways, including TCR signaling, PD-1 signaling and interferon signaling (Figure S1E).

As for immune cells, we observed that ICD scores of the Th1 subtype were the highest among all the T cell types (Figure 5C). Th1 cells were found to be positively related with the outcomes after ICB treatment [76]. Having explored the intracellular changes of ICD in T cells, we used CellPhoneDB to identify ligand–receptor pairs and molecular interactions among major T cell types (Figure S1F). The results showed extensive communications between the ICD score-high/ICD score-low T cells and other immune cells (Figure 5D). ICD score-low and ICD score-high T cells both showed higher numbers of interaction pairs with neutrophils than other immune cell types, primarily with the pair C5aR1-RPS19 and NR3C1-CXCL8 (Figure 5E). The C5aR1-RPS19 pair was found to promote tumor growth [77]. C5aR1-positive neutrophils were found to promote breast cancer glycolysis, resulting in tumor progression and poor survival [78]. Together, these results provided potential mechanisms underlying difference in survival and ICB response between ICD score-high and -low patients.

### 3.7. IGF2BP3 Regulates ICD in Colon Cancer

**Figure 6:**
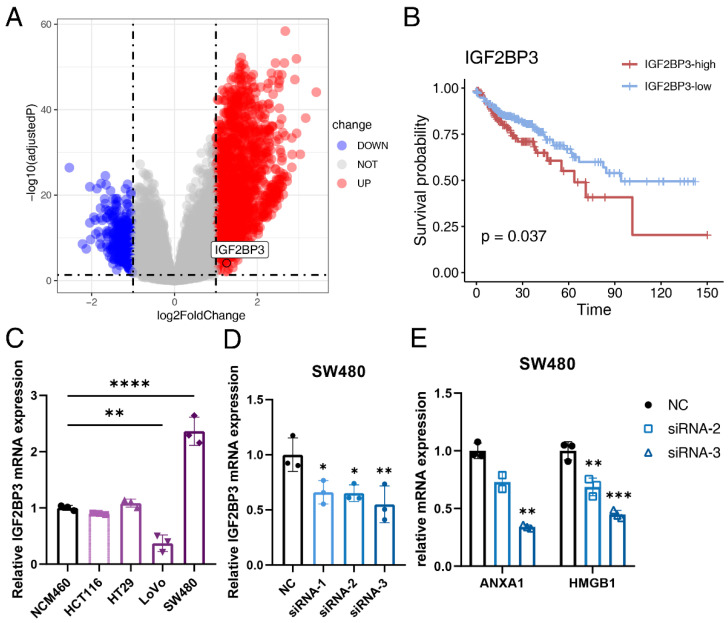
IGF2BP3 participates in the ICD process of colon cancer. (**A**) Volcano plot of differentially expressed genes (DEGs) between the high-ICD score and low-ICD score group in the TCGA-COAD cohort. Red dots represent upregulated genes, while blue dots represent downregulated genes. (**B**) Kaplan-Meier curves of OS between the IGF2BP3-high and IGF2BP3-low group. Time is measured in months and the log-rank test *p*-value is reported. (**C**) The relative expression level of IGF2BP3 in the NCM460, HCT116, HT29, LoVo and SW480 cell lines detected by quantitative real-time polymerase chain reaction (qRT-PCR). (**D**) The transfection efficiency of si-IGF2BP3 and NC in the SW480 cell line detected by qRT-PCR. (**E**) The relative expression level of ANXA1 and HMGB1 in the SW480 cell line after siRNA-2 and siRNA-3 transfection detected by qRT-PCR. Three independent experiments were carried out. * represents *p* < 0.05, ** represents *p* < 0.01, *** represents *p* < 0.001, **** represents *p* < 0.0001.

CRC is the second most common cause of cancer death in the United States [79]. Immune checkpoint inhibitors were approved by FDA for the treatment of mismatch-repair-deficient and microsatellite instability-high CRC patients [80]. However, mismatch-repair-proficient or microsatellite instability-low CRCs constitute the majority of CRC cases and these patients hardly respond to immunotherapy [81]. As ICD has synergistic effects with immunotherapy, we further identified ICD-related genes in colon cancer. Patients in the TCGA-COAD cohort were divided into the high-ICD score and low-ICD score group based on the median ICD score. DEG analysis between the high-ICD score and low-ICD score group identified 2011 significantly up-regulated genes and 381 significantly down-regulated genes (Figure 6A). Molecular pathway analysis of the DEGs using the online tool OncoboxPD revealed that numerous cancer-related pathways were regulated in different subgroups (Table S6). Further analysis of DEGs revealed 58 survival-related genes, among which Insulin Like Growth Factor 2 MRNA Binding Protein 3 (IGF2BP3) was identified (Figure 6B). IGF2BP3 negatively correlated with OS in the TCGA-COAD cohort (*p* = 0.037).

To evaluate the specific role of IGF2BP3 in ICD in colon cancer, IGF2BP3 was knocked down with siRNA. We first analyzed the relative mRNA expression level of IGF2BP3 in four human colon cancer cell lines (HCT116, HT29, SW480 and LoVo) and found that the mRNA expression level of IGF2BP3 was significantly higher in SW480 than that in the normal human colon cell line NCM460 (Figure 6C), so we chose SW480 for subsequent experiments in vitro. Transfection efficiency was evaluated by qRT-PCR and western blot. The result showed that the relative expression of IGF2BP3 at both transcriptional level (Figure 6D) and translational level (Figure S6) were all significantly down-regulated after siRNA transfection, so siRNA-2 and siRNA-3 were used for further experiments. We then analyzed the expression level of canonical ICD-related genes after knocking down IGF2BP3 and found that the relative mRNA level of ANXA1 and HMGB1 were significantly down-regulated (Figure 6E). This indicated that IGF2BP3 functioned as an inducer in the ICD process of colon cancer.

## 4. Discussion

ICD is important in cancer therapy, but comprehensive assessment of the ICD landscape at a pan-cancer level still remains unexplored. Additionally, whether ICD functions as a stimulator or a suppressor in TME remains controversial. To provide novel insights into this topic, we developed an approach for ICD state computation and revealed the connections between ICD and genomic characteristics, tumor hallmarks, immune phenotypes, clinical outcomes and therapeutic responses in different cancer types. Thus, our findings could help in better understanding ICD-related modulations in the TME and provided evidence for further investigation on ICD-based target therapy and biomarker identification. 

In this study, we developed an ICD-related prognostic model by machine learning and Cox regression analysis. We identified IL6 as an important lynchpin between ICD and cancer. Besides, we observed that higher ICD scores were associated with lower tumor purity and a higher probability of tumor metastasis and tumor hypoxia. However, we found that patients with a high ICD score were more likely to respond to ICB, which might be attributed to the low immune cell infiltration in low ICD score cancer patients. Thus, it is of great importance to develop ICD-related therapeutic targets, which might help to achieve better clinical outcomes in combination with immunotherapy.

We came up with a few solutions to solve this problem. The first is demethylation of certain ICD-related genes, like CD4 and LY96. The second solution is targeting transcriptional regulators of ICD-related genes. Regulation of Est1 with RNAi could effectively treat multidrug-resistant breast cancer [82]. The third solution is combining mTOR inhibitors with other anti-tumor therapies. mTOR inhibitors had been widely used in cancer therapies for a long time [83,84]. Whether this could stimulate ICD and result in a synergy effect with ICB warrants further experimental studies. Another solution is targeting certain receptors on immune cells. Studies have shown that C5AR1 inhibitors could impair tumorigenesis and tumor metastasis, resulting in good clinical outcomes [85,86,87,88]. Besides, application of cytokine antagonists, such as targeting the tumor-promoting cytokine IL6, might also improve therapeutic outcomes by triggering ICD in cancer patients. Actually, several cytokines or cytokine antagonists had already been studied in several clinical trials [89]. Combination use of immunotherapy with these target therapies might result in synergistic effects among immunosuppressive cancer patients.

Our study identified IGF2BP3 as a regulator of ICD in colon cancer for the first time. IGF2BP3 works as a m6A reader [90] and functions as a potential oncogene in many cancer types including colon cancer [91,92,93,94]. We found that IGF2BP3 could regulate the expression of key DAMP-related genes. Besides, we found that expression of IGFBP2 negatively correlated with OS in PCPG patients, which was consistent with previous studies which also identified IGFBP2 as an oncogene in various tumor types [95,96,97]. Altogether, these indicated that insulin-like growth factor binding protein family played an important role in the ICD tumor process.

Compared with the previous meta-analysis in lung, breast and ovarian malignancies [28], this study was carried out at a pan-cancer level, which made the relative conclusions applicable in a wider range of cancer patients. Besides, the previous study analyzed the prognostic value of each ICD-related gene separately. In the current study, the ICD-related genes were regarded as a whole and its prognostic value was validated in the TCGA cohort in combination with several important clinical characteristics. Lastly, the previous study was based solely on bulk-RNA sequencing data. In the current study, sc-RNA seq data was also analyzed to explore the relationship between ICD and cancer at a single cell resolution.

There are several limitations in our study. First, the ICD score we constructed did not always work perfectly in predicting all of the tumor characteristics and the results were sometimes not consistent across cancer types. We speculated that ICD is not a universal suppressor of tumorigenesis. Instead, its role as either an oncogene or tumor suppressor gene is context-dependent and cancer type-specific. Besides, ICD may play a double-sword role in the TME. Second, due to the limited number of patients treated with immunotherapy, we only observed significant association between ICD score and immune response in some of the cohorts tested. This relationship warrants further verification in larger cohorts, as does the application of the ICD score in clinical practice. Lastly, we only verified that IGF2BP3 could regulate the expression of ICD-related genes, but the underlying mechanisms warrant further investigation. Besides, whether targeting IGF2BP3 could increase the efficacy of immunotherapy is yet to be solved.

## 5. Conclusions

We depicted the ICD landscape in normal and cancer samples by integrating multi-omics data from various databases. We developed an ICD-related prognostic model with high predictive power. We also defined the ICD score, which proved to be a reliable marker in predicting patient prognosis and therapeutic response. However, the underlying mechanism of how ICD remodels TME is still poorly understood and warrants further investigation. These results emphasized the important role of the ICD process in tumorigenesis and tumor immunity, which will be helpful in developing ICD-related anti-cancer target treatments. This integrative study on ICD and cancer highlights a promising field for predicting prognosis and improving therapeutic outcomes in cancer patients.

## Data Availability

The datasets used and/or analyzed during the current study are available from the corresponding author upon reasonable request. The codes used for single-cell analysis can be downloaded from https://github.com/TracyWang05/ICD/tree/master, accessed on 8 October 2022.

## Supplementary Materials

The following supporting information can be downloaded at: https://www.mdpi.com/article/10.3390/cancers14235952/s1, Figure S1: Single-cell analyses reveal ICD heterogeneity and associated immune signaling; Figure S2: Landscape of ICD-related genes expression and regulators in pan-cancers; Figure S3: Clinical and functional characteristics in different ICD-subtype; Figure S4: Prognostic value of the ICD-related prognostic model; Figure S5: Associations of ICD scores with chemotherapy and immunotherapy response; Figure S6: ICD score analysis in the TCGA-COAD cohort; Table S1: Detailed information about datasets used in the study; Table S2: The whole and prognostic ICD-related genes identified by the four machine learning algorithms Boruta, XGBoost, SVM and Random Forest; Table S3: Cell markers used for cell type annotation; Table S4: Sequences of siRNAs and quantitative real-time polymerase chain reaction (qRT-PCR) primers; Table S5: Results of the transcriptional regulators of the ICD related genes predicted by iRegulon; Table S6: PALs of DEGs between the high-ICD score group and the low-ICD score group in the TCGA-COAD cohort.
