# Supplementary material for: Comprehensive Pan-Cancer Analyses of Immunogenic Cell Death as a Biomarker in Predicting Prognosis and Therapeutic Response
Source: Cancers (Basel). 2022 Dec 1;14(23):5952. doi: 10.3390/cancers14235952 (PMC9736000; doi:10.3390/cancers14235952)
Supplement: Supplementary file 1 [file cancers-14-05952-s001.zip › cancers-1988425-Supplementary material.pdf]

Comprehensive pan-cancer analyses reveal the value of immunogenic cell death as a biomarker in predicting prognosis and therapeutic response

Supplementary material

Figure S1 Single-cell analyses reveal ICD heterogeneity and associated immune signaling. **(A)** UMAP of 30,642 high-quality cells was captured from the scRNA-seq data, colored by the cluster information and three main cell types. **(B)** UMAP plot showing eleven sub-clusters of epithelial cells. **(C)** UMAP plot showing eighteen subtypes of immune cells, colored by clusters and cell types. **(D)** UMAP plot showing seven sub-clusters of T cells. **(E)** Plot showing significant enriched Reactome pathways of DEGs between the epithelial cells in the high-ICD score and low-ICD score group. **(F)** Capacity for intercellular communication between immune cells. Each line color indicates the ligands expressed by the cell population represented in the same color (labeled). The lines connect to the cell types that express the receptors. The line thickness is proportional to the number of ligands when receptors are present in the recipient cell type. The loops indicate autocrine circuits.

Figure S2 Landscape of ICD-related genes expression and regulators in pan-cancers. **(A)** A comprehensive heatmap illustrating the expression pattern of ICD-related genes across 26 cancer cell types from CCLE. **(B)** The Spearman correlation analysis was performed among all 34 ICD-related genes in pan-cancers. **(C)** Heatmap showing the correlation between the methylation level and transcription level of ICD-related genes in pan-cancers. **(D)** The master regulators predicted by the iRegulon tool are highlighted in yellow and target genes in green. * represents FDR < 0.05, **

represents FDR < 0.01, *** represents FDR < 0.001.

Figure S3 Clinical and functional characteristics in different ICD-subtypes. **(A)** Average ICD scores across normal tissues in the GTEx dataset. **(B)** Differentially expressed proteins between the ICD-CB subtype and the ICD-CC subtype across TCGA cancer types.

Figure S4 Prognostic value of the ICD-related prognostic model. Feature selection using the machine learning algorithms Boruta **(A)**, XGBoost **(B)**, and Random Forest **(C)**. **(D)** Univariate cox regression analysis of OS of each ICD-related gene identified by machine learning and important clinical characteristics in the TCGA cohort. **(E)** Multivariate cox regression analysis of OS of the predictors included in the ICD-related prognostic model in the TCGA cohort. **(F)** Kaplan–Meier survival curve of OS between patients stratified by the ICD-related prognostic model in the TCGA cohort. **(G)** The time-dependent ROC of the ICD-related prognostic model at 5, 10, and 20 year in the TCGA cohort. **(H)** Kaplan–Meier survival curve of OS between patients stratified by the ICD-related prognostic model in the ICGC cohort. **(I)** The time-dependent ROC of the ICD-related prognostic model at 5, 10, and 20 year in the ICGC cohort. * represents p < 0.05, ** represents p < 0.01, *** represents p < 0.001.

Figure S5 Associations of ICD scores with chemotherapy and immunotherapy response. **(A)** Spearman correlation analysis between ICD scores and IC50 of 198 compounds from GDSC in pan-cancers. **(B)** Boxplot showing the TMB of the ICD-CA, ICD-CB, and ICD-CC subtype. **(C)** Total mutation rates of seven DDR pathways were summarized in the ICD-high and -low group across cancers. **(D)** ROC curves of

the ICD score in distinguishing responders from non-responders in two immunotherapy cohorts. The AUC for each cohort was shown.

Figure S6 Transfection efficiency of si-IGF2BP3 and NC in the SW480 cell line detected by western blot. Three independent experiments were carried out. **** represents $p < 0.0001$.

Table S1 Detailed information about datasets used in the study.

Table S2 The whole and prognostic ICD-related genes identified by the four machine learning algorithms Boruta, XGBoost, SVM, and Random Forest.

Table S3 Cell markers used for cell type annotation.

Table S4 Sequences of siRNAs and quantitative real-time polymerase chain reaction (qRT-PCR) primers.

Table S5 Results of the transcriptional regulators of the ICD-related genes predicted by iRegulon.

Table S6 PALs of DEGs between the high-ICD score group and the low-ICD score group in the TCGA-COAD cohort.
