# Supplementary figures and images for: Comprehensive Pan-Cancer Analyses of Immunogenic Cell Death as a Biomarker in Predicting Prognosis and Therapeutic Response
Source: Cancers (Basel). 2022 Dec 1;14(23):5952. doi: 10.3390/cancers14235952 (PMC9736000; doi:10.3390/cancers14235952)

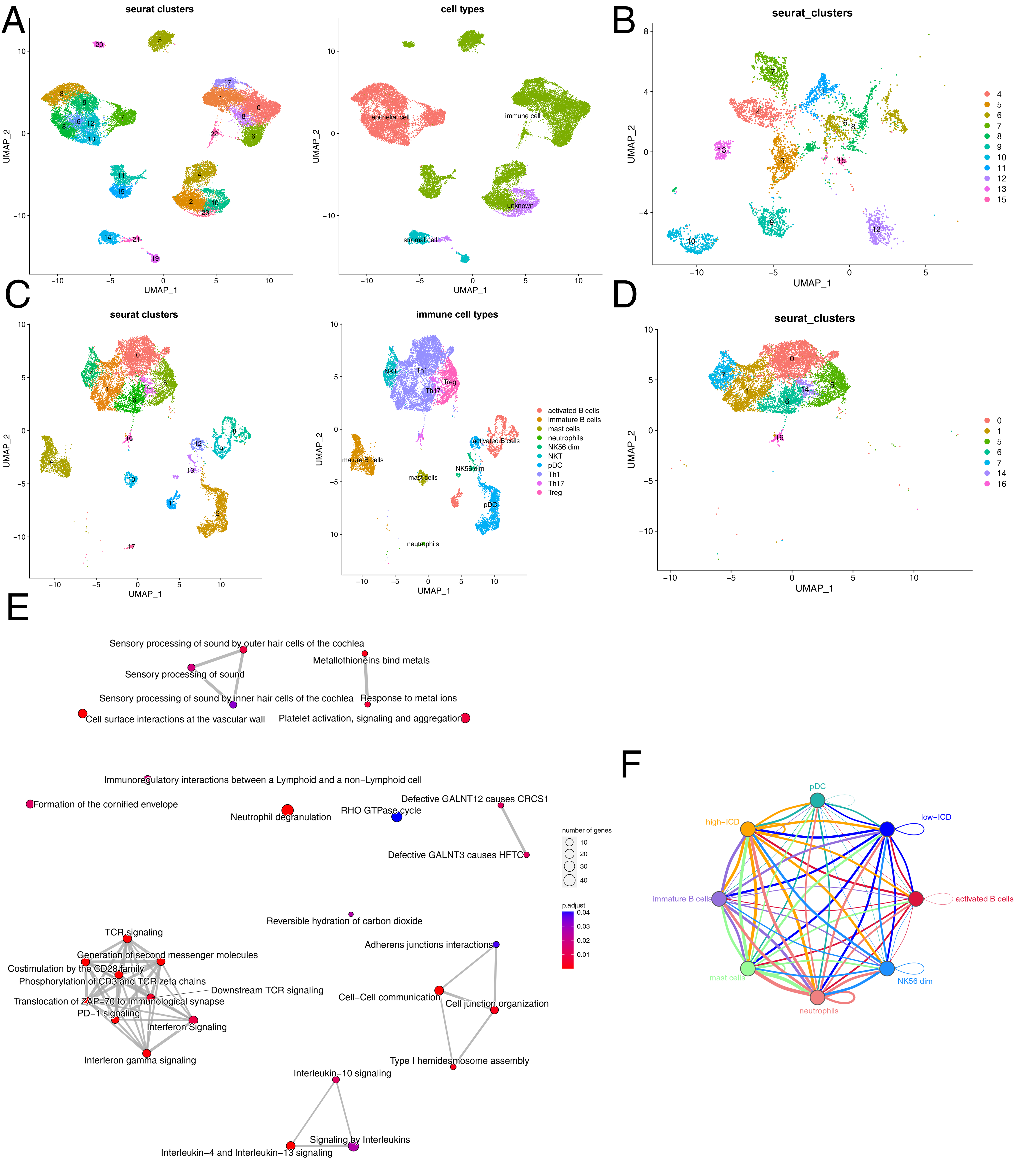

Supplement: Supplementary file 1 [file cancers-14-05952-s001.zip › Figure S1.tif]

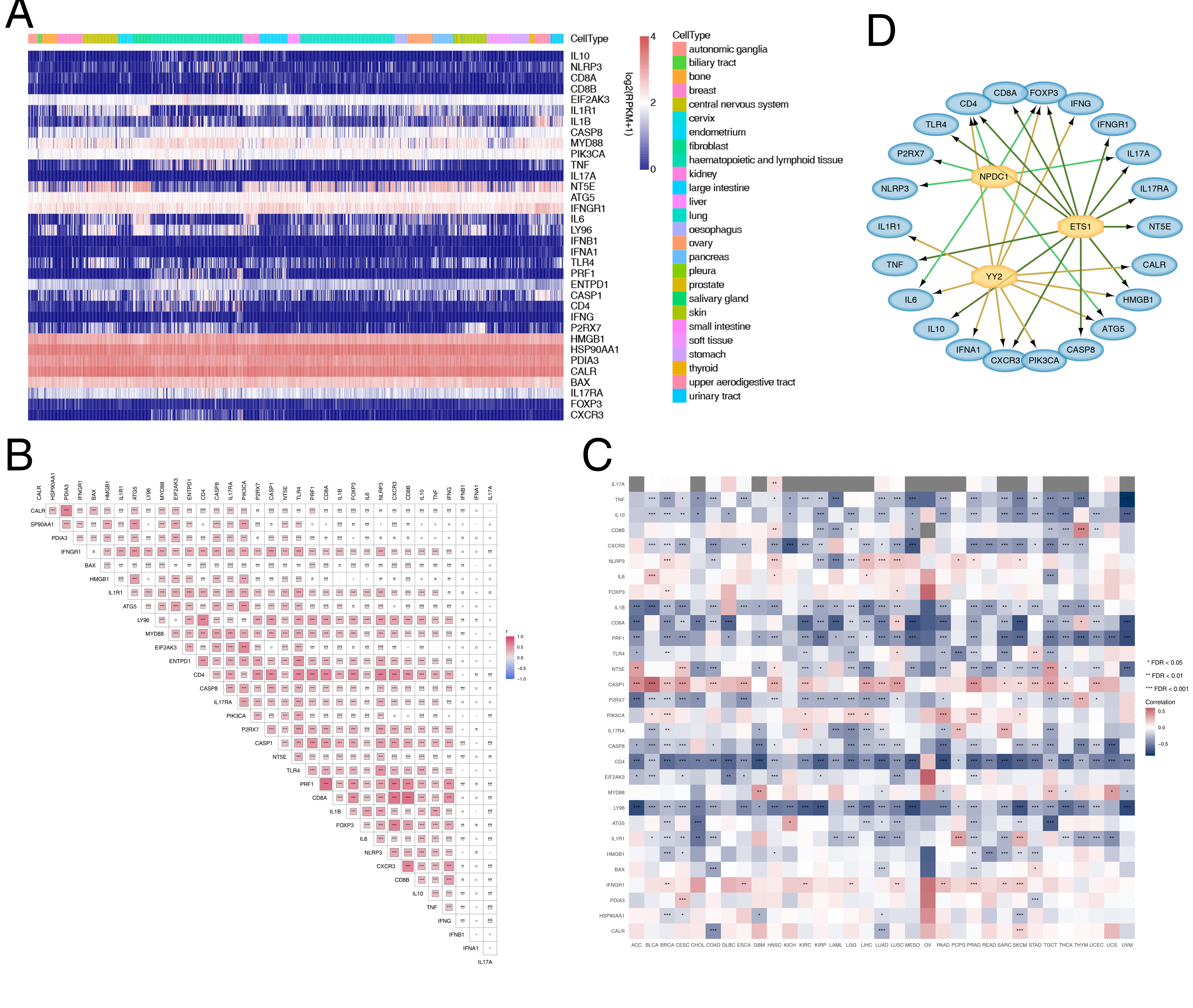

Supplement: Supplementary file 1 [file cancers-14-05952-s001.zip › Figure S2.tif]

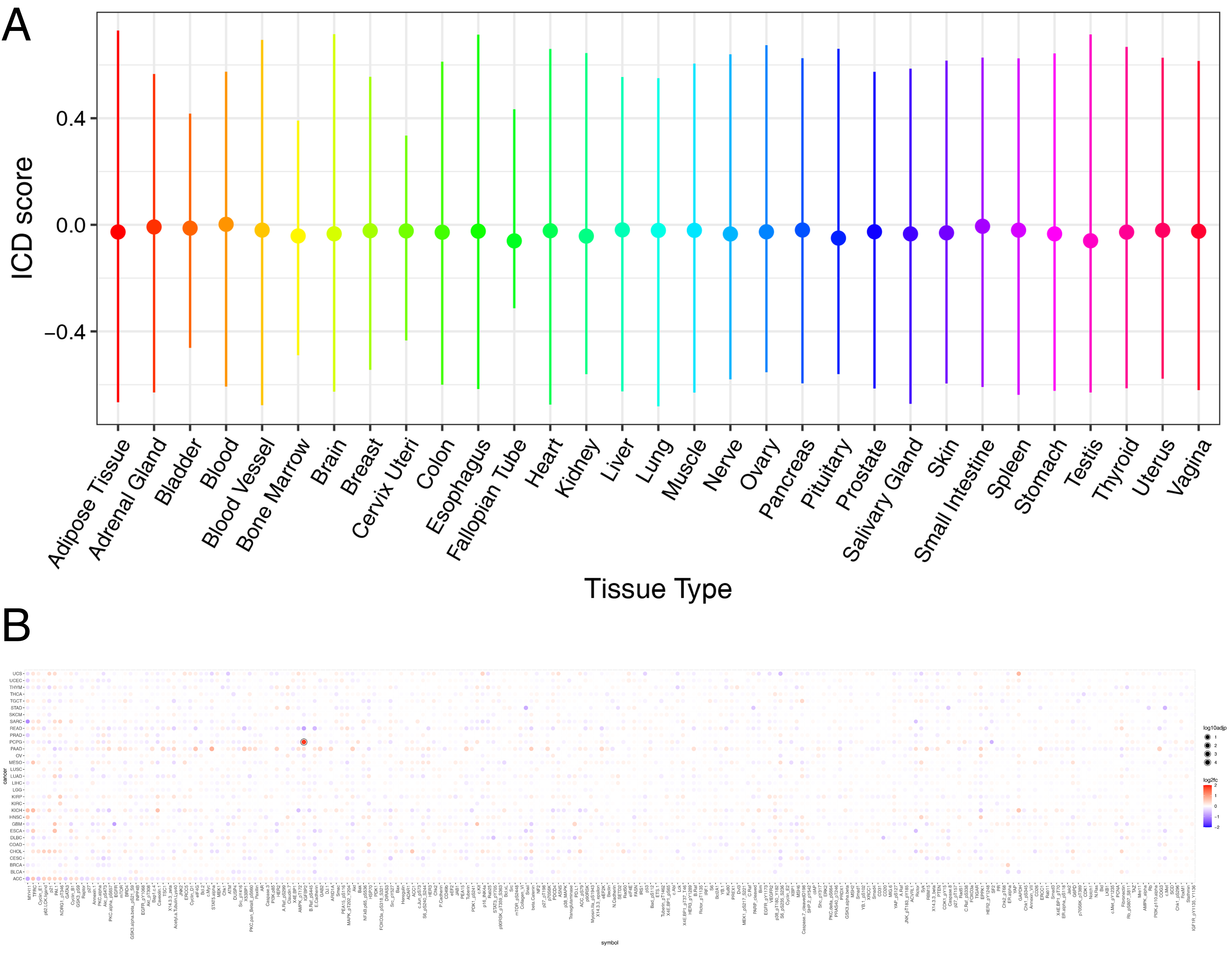

Supplement: Supplementary file 1 [file cancers-14-05952-s001.zip › Figure S3.tif]

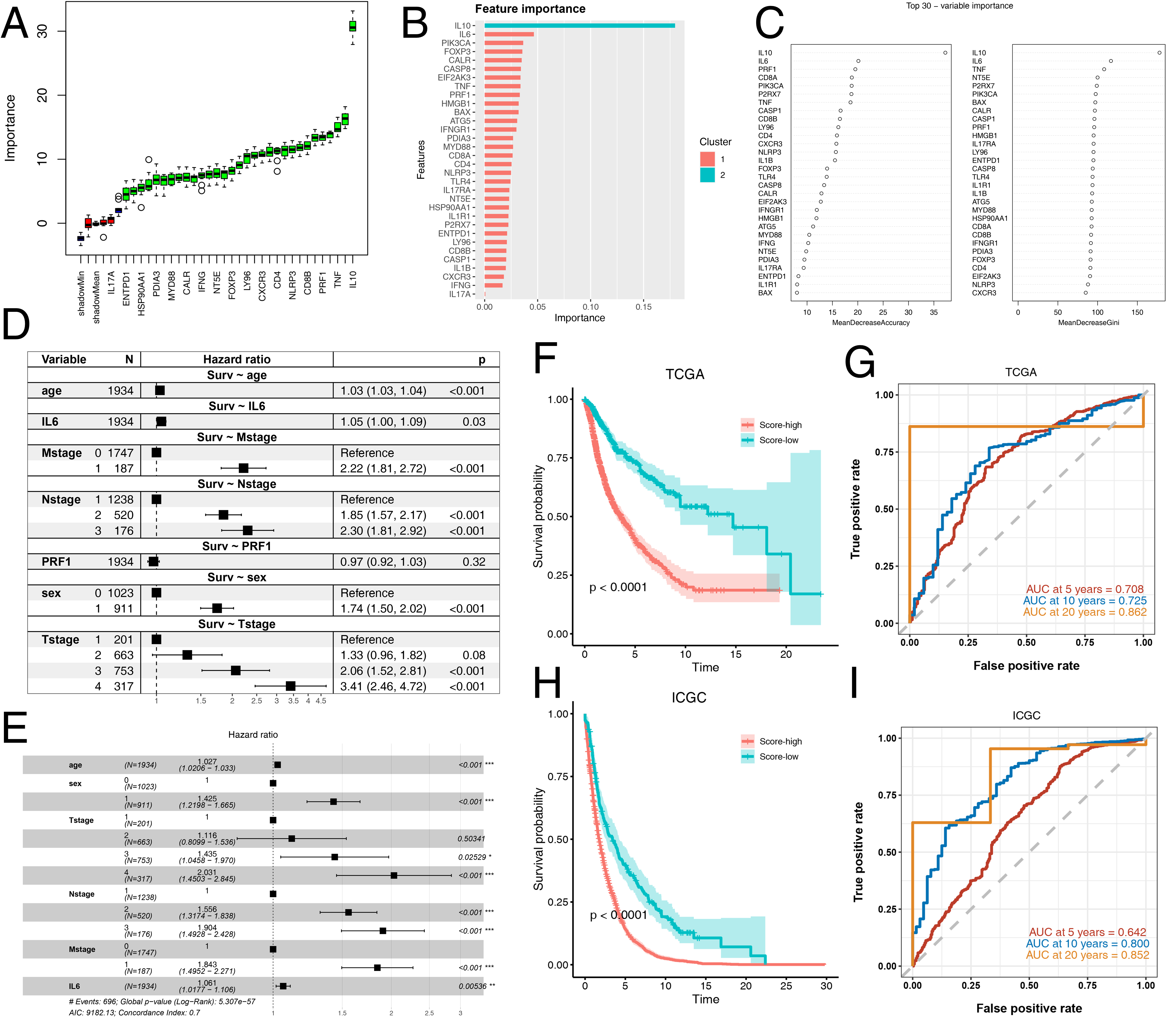

Supplement: Supplementary file 1 [file cancers-14-05952-s001.zip › Figure S4.tif]

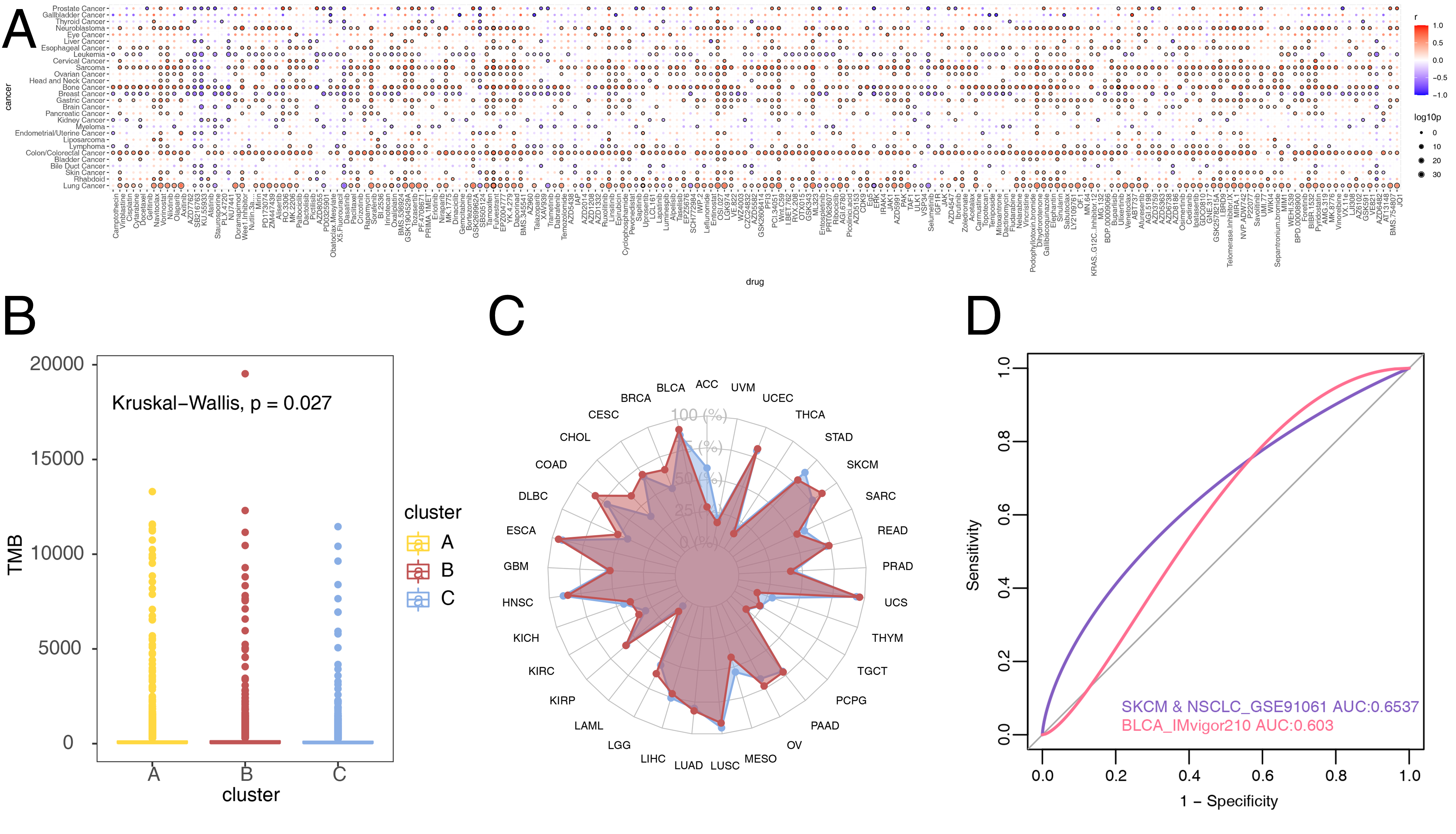

Supplement: Supplementary file 1 [file cancers-14-05952-s001.zip › Figure S5.tif]

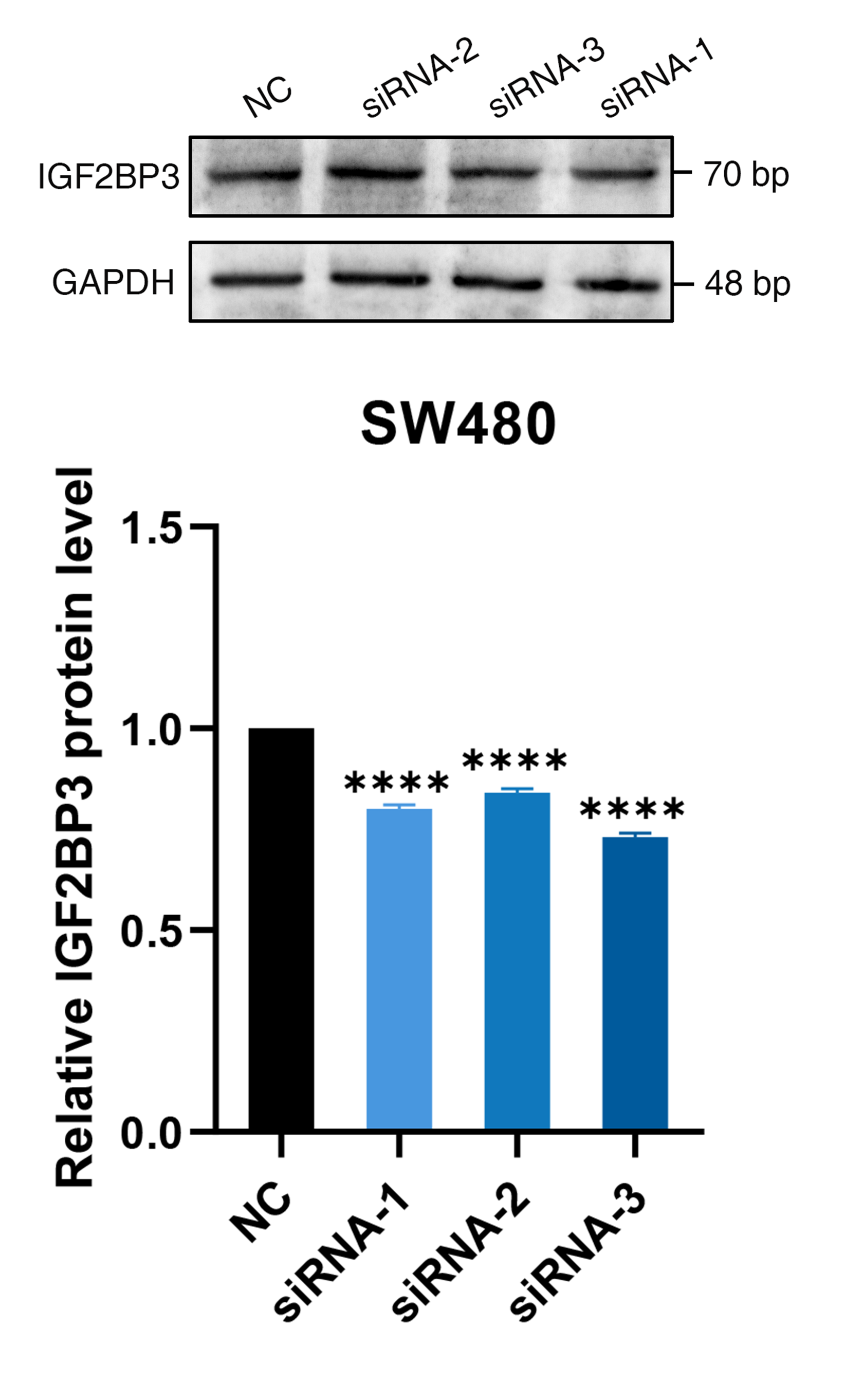

Supplement: Supplementary file 1 [file cancers-14-05952-s001.zip › Figure S6.tif]
